# Habit

**Published:** 1874-02

**Authors:** 


### HABIT.

BURKE relates that for a long time he had been under the necessity of frequenting a certain place every day, and that, so far from finding a pleasure in it, he was affected with a sort of uneasiness and disgust; and yet, if by any means he passed by the usual time of going thither, he felt remarkably uneasy, and was not quieted until he was in his usual track.

Persons who use snuff soon deaden the sensibility of smell, so that a pinch is taken unconsciously, and without any sensation being exerted thereby, sharp though the stimulus may be.

After a series of years winding up a watch at a certain hour, it becomes so much a routine as to be done in utter unconsciousness; meanwhile the mind and body are engaged in something entirely different.

An old man is reported to have scolded his maid-servant very severely for not having placed his glass in the proper position for shaving. "Why, sir," replied the girl, "I have omitted it for months, and I thought you could shave just as well without it."

We are all creatures of habit, and the doing of disagreeable things may become more pleasant than omissions; showing to the young the importance of forming correct habits in early life, to the end that they may be carried out without an effort, even although at first it may have required some self-denial, some considerable resolution to have fallen into them.

But if doing disagreeable things does by custom become more pleasurable than their omission, then the doing right, because we love to do what is right, becomes a double pleasure to the performer in the consciousness that, while he is yielding allegiance to his Maker, he benefits his fellow-man, and can not get out of the habit of well-doing without an effort and a pang. Thus are the truly good hedged round about, and are more confirmed in their good doing, and its practice becomes easier and more delightful the longer they live, helping them to go down to the grave "like as a shock of corn cometh in his season."

But if there is something in the fixedness of good habits that binds us to them, there is the same thing as to the evil. Thus it is that when a man has arrived at the age of forty-five years, he seldom changes his opinions or his practices, which, if they are evil, become more and more fixed. Thus, what a man believes and practices at forty-five, he is likely to believe and practice till he dies; and there is small hope of his conversion to different views and different deeds, and the Ethiopian's skin, or the leopard's spots are his forever. The man, therefore, who is not a Christian — by principle, and profession, and practice — at that age, should regard his condition "with fear and trembling," for it is most likely that he never will be one.

These principles are equally applicable to our physical nature—to bodily health. Habits of regularity, temperance, cleanliness, and exercise become a second nature in the course of years; their performance a pleasure, their infraction a discomfort; while the use of beverages of ale, beer, cordials, cider, and other drinks containing alcohol, or the employment of tobacco in chewing, smoking, or snuffing, and the over-indulgence of the propensities, becomes a slavery, an iron despotism, which in the end debases the heart, undermines the health, and destroys life, making a miserable wreck of soul, body and estate together.

